# A parietal region processing numerosity of observed actions: An FMRI study

**DOI:** 10.1111/ejn.14930

**Published:** 2020-08-10

**Authors:** Hiromasa Sawamura, Burcu A. Urgen, Daniele Corbo, Guy A. Orban

**Affiliations:** ^1^ Department of Medicine and Surgery University of Parma Parma Italy; ^2^ Department of Ophthalmology the University of Tokyo Graduate School of Medicine Tokyo Japan; ^3^ Department of Psychology Bilkent University Ankara Turkey; ^4^ Interdisciplinary Neuroscience Program Bilkent University Ankara Turkey; ^5^ Aysel Sabuncu Brain Research Center and National Magnetic Resonance Research Center Bilkent University (UMRAM) Ankara Turkey; ^6^ Neuroradiology Unit Department of Medical and Surgical Specialties Radiological Sciences and Public Health University of Brescia Brescia Italy

**Keywords:** action observation, human fMRI study, numerosity of actions, posterior parietal cortex

## Abstract

When observing others' behavior, it is important to perceive not only the identity of the observed actions (OAs), but also the number of times they were performed. Given the mounting evidence implicating posterior parietal cortex in action observation, and in particular that of manipulative actions, the aim of this study was to identify the parietal region, if any, that contributes to the processing of observed manipulative action (OMA) numerosity, using the functional magnetic resonance imaging technique. Twenty‐one right‐handed healthy volunteers performed two discrimination tasks while in the scanner, responding to video stimuli in which an actor performed manipulative actions on colored target balls that appeared four times consecutively. The subjects discriminated between two small numerosities of either OMAs (“Action” condition) or colors of balls (“Ball” condition). A significant difference between the “Action” and “Ball” conditions was observed in occipito‐temporal cortex and the putative human anterior intraparietal sulcus (phAIP) area as well as the third topographic map of numerosity‐selective neurons at the post‐central sulcus (NPC3) of the left parietal cortex. A further region of interest analysis of the group‐average data showed that at the single voxel level the latter area, more than any other parietal or occipito‐temporal numerosity map, favored numerosity of OAs. These results suggest that phAIP processes the identity of OMAs, while neighboring NPC3 likely processes the numerosity of the identified OAs.

## Graphical abstract

When compared the “numerosity of observed manipulative actions” condition to “numerosity of colors of balls” condition, statistical parametric maps revealed a significant activation in the 3rd topographic map of numerosity‐selective neurons at the post‐central sulcus (NPC3, black diamond) of the left parietal cortex in addition to regions known as the action observation network. Further single voxel analysis suggested that NPC3 likely processes the numerosity of the identified observed actions.

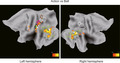

**Abbreviations**
2‐AFC: two‐alternative forced‐choice
AIP: anterior intraparietal
DIPSA: dorsal intraparietal sulcus anterior
DIPSM: dorsal intraparietal sulcus medial
fMRI: functional magnetic resonance imaging
IPS: intraparietal sulcus
LOTC: lateral occipito‐temporal cortex
MTG: middle temporal gyrus
NF: numerosity frontal map
NPC1: first numerosity postcentral sulcus map
NPC2: second numerosity postcentral sulcus map
NPC3: third numerosity postcentral sulcus map
NTO: numerosity temporo‐occipital map
OA: observed action
OMA: observed manipulative action
OTS: occipito‐temporal sulcus
phAIP: putative human anterior intraparietal sulcus
PPC: posterior parietal cortex
ROI: region of interest
SPM: statistical parametric map

## 1 INTRODUCTION

Social interactions belong to the core of human behavior, underscoring the importance of perceptual mechanisms processing visual signals arising from others' actions (Platonov & Orban, 2016, 2017). Considerable evidence points to the involvement of regions in lateral occipito‐temporal cortex (LOTC), posterior parietal cortex (PPC), and premotor cortex in action observation (Caspers, Zilles, Laird, & Eickhoff, 2010; Cross, Kraemer, Hamilton, Kelley, & Grafton, 2009; Jastorff, Begliomini, Fabbri‐Destro, Rizzolatti, & Orban, 2010; Lingnau & Downing, 2015; Wurm & Lingnau, 2015). Categorical distinctions of observed actions (OAs) have been found in LOTC, in particular the abstract action categories transitivity and sociality (Wurm, Caramazza, & Lingnau, 2017), and more recently, the action components such as body parts, scenes, movements, objects, sociality, and transitivity (Tucciarelli, Wurm, Baccolo, & Lingnau, 2019). On the other hand, recent evidence (Lanzilotto et al., 2019, 2020) indicates that PPC regions process the visual identity of OAs, in a similar way to the processing by the ventral pathway of the visual identity of objects (Hung, Kreiman, Poggio, & DiCarlo, 2005), and in particular of faces (Chang & Tsao, 2017). By visual identity of OAs, we refer to the integration of the goal of the action, that is, the change in the outside world it aims to produce, and the body movements of the conspecific that allow this goal to be reached. Thus, OA identity, as it is used here, is a purely visual notion that applies in first instance to conspecifics, and is very different from the abstract concepts of action verbs that may apply to many living creatures and even objects (e.g., running water). Furthermore, it does not include the actor, nor the object, target of the action, nor the scene (Orban, 2018). Recent findings suggest that OA identity may be encoded at two levels in PPC: an aerial level for OA classes and a single neuron level for OA exemplars. Indeed, different classes of OAs, such as manipulative actions, locomotive actions, or skin displacing actions, are processed in different PPC regions (Abdollahi, Jastorff, & Orban, 2013; Corbo & Orban, 2017; Ferri, Rizzolatti, & Orban, 2015). On the other hand, the exemplars of a given class are processed by OA selective neurons, as has been shown for observed manipulative actions (OMAs) in monkey anterior intraparietal (AIP) area (Lanzilotto et al., 2019) and its human homologue, putative human anterior intraparietal sulcus (phAIP; Aflalo et al., 2020). The link between phAIP and the processing of OMAs identity is further supported by the activation of this region in two‐alternative forced‐choice (2AFC) discrimination tasks with OMAs as discriminanda (Orban, Ferri, & Platonov, 2019; Platonov et al., 2020). It has been proposed that the processing of visual identity of OA is one of the main signals used by subjects in the planning of own actions (Lanzilotto et al., 2019), which very frequently are made in response to what others do (Newman‐Norlund, van Schie, van Zuijlen, & Bekkering, 2007). This relevance for action planning, which is common to monkeys and humans, is the primary reason why the OA identity is processed at the PPC level. During the hominin evolution, the perceptual aspect of OA identity may have gained more relevance and, at least in humans, provided input to the semantic system. Visual signals related to what others are doing, however, may not be the only signals required for planning manipulative actions. Visual signaling of how frequently conspecifics perform a given manipulative action may also be needed. For example, when learning by imitation, it may be important for the pupil to observe how many times the master repeated a given action to obtain the desired result. Conversely, observing that a conspecific repeated the same action several times with little result may provide a strong impetus to plan a different action that may prove more efficient. Hence, one may postulate the existence of a PPC region processing the numerosity of OAs. In particular, a PPC region receiving OMA identity signals from phAIP may process the visual signals further to extract OMA numerosity.

Which PPC regions might be involved in the processing of OMA numerosity? One lead might be provided by the regions involved in processing numerosity of executed actions, as action observation and execution often engage similar regions (Filimon, Nelson, Hagler, & Sereno, 2007; Iacoboni et al., 1999; Lanzilotto et al., 2019; Maeda, Ishida, Nakajima, Inase, & Murata, 2015; Nelissen & Vanduffel, 2011; Pani, Theys, Romero, & Janssen, 2014). In the monkey, neurons selective for the number of turn or push arm movements performed by the monkey have been reported some time ago in the medial wall of the intraparietal sulcus (IPS) (Sawamura, Shima, & Tanji, 2002, 2010). It is unclear however what human region corresponds to this monkey parietal region. An alternative lead might be provided by recent high‐field functional magnetic resonance imaging (fMRI) experiments, revealing the topographical mapping of numerosity‐selective neurons in the human cortex (Harvey, Klein, Petridou, & Dumoulin, 2013). The set of numerosity maps revealed by these studies included a numerosity temporo‐occipital (NTO) map at the temporo‐occipital junction, a numerosity parieto‐occipital map in the parieto‐occipital sulcus, three numerosity postcentral maps (NPC1‐3) along the IPS, and a numerosity frontal map at the junction of the precentral and superior frontal sulci (Harvey & Dumoulin, 2017). Particularly, two of these maps, NPC2 and NPC3, located in the immediate vicinity of phAIP (Harvey, Ferri, & Orban, 2017), are likely candidates for processing OMA numerosity. The present study was set up to test this hypothesis and investigate the role of NPC2 and NPC3 in the processing of OMA numerosity.

To reach that aim, we employed an attentional modulation paradigm in which featural attention to a constant stimulus was manipulated (Cant & Goodale, 2007; Chiu, Esterman, Han, Rosen, & Yantis, 2011; Orban et al., 2019; Peuskens et al., 2004). Subjects attended to the numerosity of either OMAs or the colored targets of those actions, while viewing identical video clips, and performed 2AFC tasks, discriminating either the number of OAs or of colored targets. Two closely related factors were critical in solving the current tasks: (a) featural attention devoted to OAs or color of targets and (b) numerosity of the attended features. Thus, the tasks are well suited to reveal the cortical areas processing the identity of OAs and the numerosity of the identified actions. We tested for an interaction at the random‐effects group level between the factors numerosity and attended feature in the brain regions of interest. In addition, in an exploratory analysis, we also examined in a number of region of interest (ROIs) the single voxel activity for this interaction.

## 2 MATERIALS AND METHODS

### 2.1 Subjects

Twenty‐six right‐handed (evaluated by self‐report), healthy volunteers with normal or corrected‐to‐normal visual acuity participated in the fMRI study. Among these subjects, three were eliminated because of excessive head motion (the head moved more than 1 mm in more than 15% of volumes in each run) and two were eliminated because of low accuracy in the task (<85%, i.e., more than 2 *SD*s (4.74%) from the mean (95.4%). In total, 21 subjects (six males; mean ± *SD* age, 26.0 ± 3.1 years; range, 21–34 years) contributed to the current experiment. Subjects were naive to the purpose of the experiment and provided informed consent for participation. The Ethics Committee of the Province of Parma approved this study. Experiments were performed in accordance with the national and European guidelines for testing human subjects.

### 2.2 Visual stimuli

**FIGURE 1:**
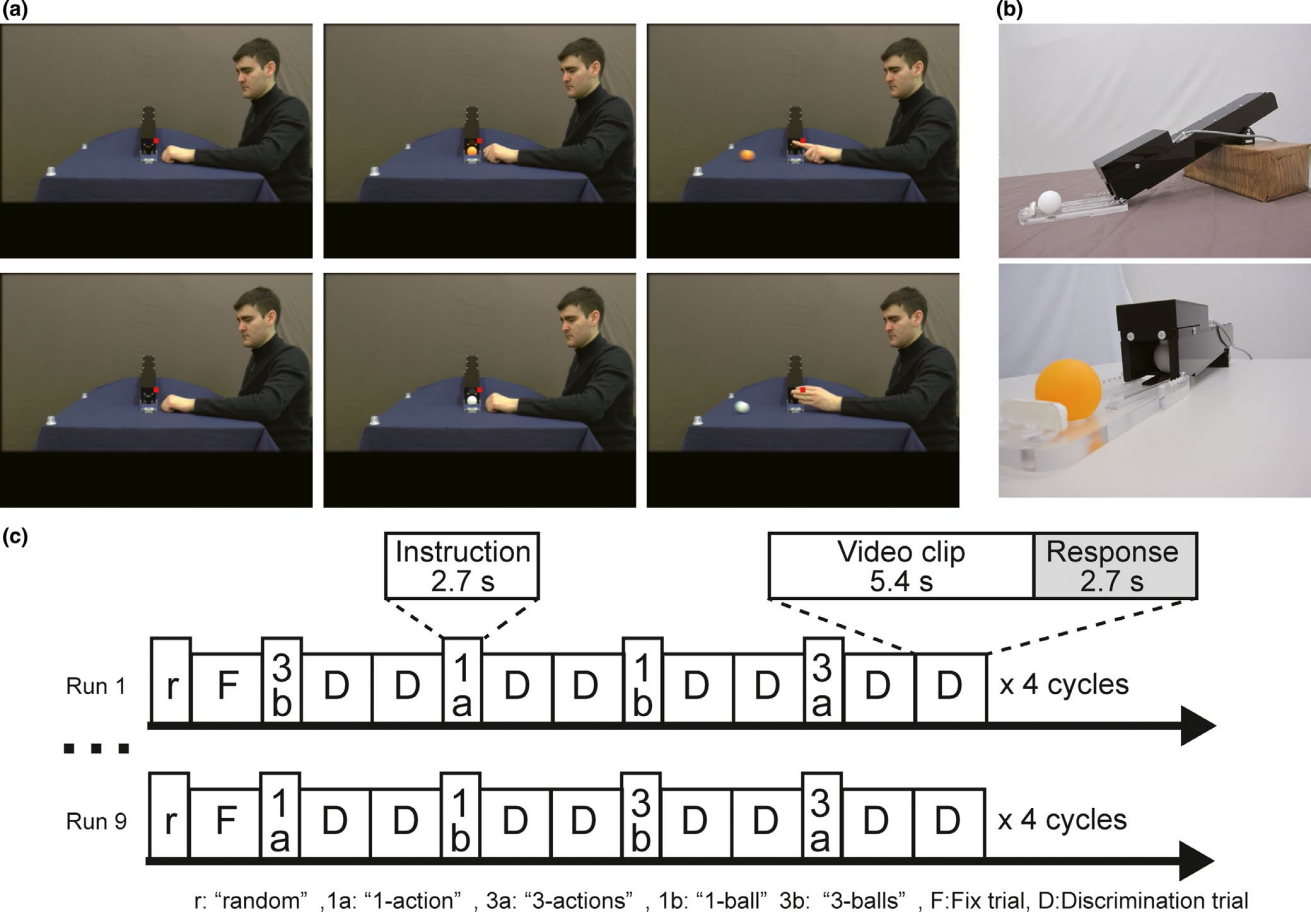
(a) Examples of three frames taken from the video clips used as visual stimuli: from left to right: before the target ball appearance, appearance of the target ball, and immediately after performance of the action. Upper row presents flicking an orange ball with the index finger; lower row presents pushing a white ball with the hand. Red square point indicates the fixation target. (b) The lateral view of the device ejecting the balls in the upper panel and enlarged frontal view of the device in the lower panel. At the outlet, the next white ball is kept until the electrically controlled switch allows the ball to roll down. (c) Time course of each run. A set of instructions was presented for 2.7 s, followed by two discrimination trials. Each discrimination trial consisted of 5.4‐s video clips, presenting one of four combinations of actions and their targets, and a 2.7‐s response period

Each run included two types of trials: discrimination and fixation trials. Each discrimination trial consisted of a 5.4‐s video clip (17 × 13 degrees, 50 frames per second), followed by 2.7‐s response period. Video edges were blurred whereby the video clip gradually faded into the black background, thus avoiding sharp contrasting borders between video and background. Video clips in discrimination trials showed a male or female actor sitting at the right side of a table and performing an action on a colored ping‐pong ball, which was ejected from a black mechanical device positioned on the back of the table (Figure 1a). The device could hold six balls inside the tube and was tilted sufficiently to make a ball roll down the ramp (Figure 1b). The gate was electrically controlled allowing precise timing of the appearance of the balls. In the video clips, four white or orange balls were automatically ejected from the black device sequentially at 1.2‐s intervals and rolled toward the front of the table. The ratios of white to orange balls were either 1:3 or 3:1. The color of the successive balls was unknown to the observers until balls appeared at the outlet of the instrument. The actor performed either a push or flick motor act (ratio of 1:3 or 3:1) on the four balls to aim for the goal between two gates on the left side of the table (opposite the actor). To do this, the actor either moved the whole hand away from him in pushing or extended his index finger as a spring in flicking, to alter the trajectory of the balls by approximately 90° (from rolling forwards to rolling to the left). The actor made a fist on the table until he/she acted on the balls; thus, the action was unknown to the observer until the actor performed it on the balls. As the hand was positioned close to the trajectory of the balls, the actor used predominantly his/her fingers and hand to manipulate the balls. The motor act performed by the actor and the color of the ball ejected were combined pseudo‐randomly. As a fixation target, a small red square appeared in the center of the screen, immediately above or below (50% each) the actor's hand to allow subjects to use their upper or lower visual fields to observe the actions, to avoid vertical asymmetries in visual field stimulation. In one‐half of the trials, the videos were flipped such that the actor appeared on the left side of the table (and the goal for the ball on the right), to avoid left‐right asymmetries in visual field stimulation. During the response period, a blank screen of average luminance, the same size as that of the video clip, was presented with a small green square in its center as a fixation point. In fixation trials, a blank screen of average luminance with a red small square in the center of the screen was presented for 5.4 s. The change of color of the fixation target from red to green signaled to the subjects the start of the response period.

### 2.3 Task

One of four different instructions (“1‐action,” “3‐actions,” “1‐ball,” and “3‐balls”) was presented for 2.7 s in Italian with yellow bold characters against a background of same average luminance as the video clip, followed by two consecutive discrimination trials of the type indicated. The instruction “random” appeared for 2.7 s, to announce a fixation trial (Figure 1c). In the four experimental conditions, subjects were required to watch the videos, maintain fixation on a small colored square, and perform the 2AFC discrimination task. The subjects discriminated the number of motor acts in the video (i.e., which action, pushing or flicking, was performed one time in the block of “1‐action” or three times in the block of “3‐actions”) or number of color balls (i.e., which colored ball, white or orange, appeared one time in the block of “1‐ball” and three times in the block of “3‐balls”), and responded by pressing one of two buttons with the index or middle finger of the right hand during the response period. Depending on the task, these two fingers corresponded to push/flick or white/orange. During the fixation trials, subjects made random choices and responses. We defined three conditions: “Action” condition including discrimination blocks of “1‐action” and “3‐actions”; “Ball” condition including discrimination blocks of “1‐ball” and “3‐balls”; and “Fix” condition including fixation trials.

Data were collected in a single nine‐run session. One run consisted of four cycles, each including a fixation trial and the four different discrimination blocks, “1‐action,” “3‐actions,” “1‐ball,” and “3‐balls,” with two discrimination trials included in each of the blocks (Figure 1c). Each discrimination block was pseudo‐randomly presented within a cycle and counterbalanced across runs and participants. Two numbers of actions (three or one) could be performed by a male or female actor on two numbers of balls (three or one), with two positions of the actor in the visual field (right or left). Moreover, three different orders of appearance of actions (e.g., pushing, flicking, pushing, and pushing; or pushing, flicking, flicking, and flicking) and three different orders of appearance of colored balls (e.g., white, white, orange, and white; or orange, orange, orange, and white) were prepared to avoid prediction by subjects. Thus, 2 × 2 × 2 × 2 × 3 × 3 = 144 different videos were generated (Table 1). From these 144 videos, 72 were selected to maintain equal presentation of actor gender, numbers of pushing and flicking actions, numbers of white and orange balls, and actor position. Each of the 72 videos appeared once in a block, as the same videos were repeated in the four cycles of a run. Thus, visual stimuli were identical in the four discrimination blocks “1‐action,” “3‐actions,” “1‐ball,” and “3‐balls.”

**TABLE 1:** Factors randomized in the experiment

Action	Gender	Target	Actor's position	Order of actions	Order of balls
Push/Flick	Male/Female	Orange/White	Upper‐right/Lower‐left	3 different orders	3 different orders

### 2.4 FMRI data acquisition, preprocessing, and analysis

We employed procedures similar to those of Orban et al. (2019) for data collection and analysis. Briefly, before the scanning session, all subjects were trained using visual stimuli different from those in the scanning session. The structure of the training session was similar to that of the scanning session, except for the provision of auditory feedback after each response, informing subjects that the response was correct or incorrect. The training procedure was repeated twice outside the scanner and twice inside the scanner. In the scanner, visual stimuli were presented in the fronto‐parallel plane by means of a head‐mounted display (60‐Hz refresh rate) with a resolution of 800 × 600 pixels (Resonance Technology) in each eye. The display was controlled by an ATI Radeon 2400 DX dual‐output video card (AMD), driven by E‐prime software (Psychology Software Tools). To reduce head motion, each subject's head was restrained with cushions. Subjects indicated responses by pressing a button under the index or middle finger using a response box (Resonance Technology) positioned under the right hand. Throughout the scanning session, eye movements were recorded with an infrared eye‐tracking system (60 Hz; Resonance Technology).

Scanning was performed in the Hospital of Parma using a 3T MR scanner (GE Discovery MR750) with an eight‐parallel‐channel receiver coil. Functional images were acquired using gradient‐echoplanar imaging with the following parameters: 49 horizontal slices (2.5 mm slice thickness; 0.25 mm gap), repetition time (TR) = 2.7 s, time of echo (TE) = 21 ms, flip angle = 90°, 96 × 96 matrix with field of view = 240, and ASSET = 2. A 3D T1‐weighted IR‐prepared fast SPGR (Bravo) image was acquired and used for anatomical reference with these parameters: TE/TR 3.7/9.2 ms; inversion time = 650 ms; flip angle = 128; ARC = 2; and 186 sagittal slices acquired with 1 × 1 × 1 mm^3^ resolution.

Data analysis was performed with the SPM8 software package (Wellcome Department of Cognitive Neurology). Preprocessing procedures involved (a) realignment, (b) co‐registration of anatomical and mean functional images, (c) spatial normalization to standard MNI152 space (by estimating the optimum 12‐parameter affine transformation and non‐linear deformation with a voxel size of 2 × 2 × 2 mm), and (d) smoothing (isotropic Gaussian kernel of 6 mm). We next applied to the nine runs a generalized linear model composed of nine regressors: three for two experimental conditions (“Action,” “Ball”) and the control condition (Fix) and six for motion parameters. This model was extended to 11 regressors (five experimental and six motion parameters) when considering the action conditions separately. Condition‐specific regressors were convolved with the canonical hemodynamic response function (HDR).

Three contrasts were defined at the subject level: Action condition versus Ball condition, Action condition versus Fix condition, and Ball condition versus Fix condition. An average statistical parametric map (SPM) was generated at the second, random‐effects level with the FWE‐corrected threshold of *p* < .05. In addition, the same procedure was used to compare the two action conditions directly.

### 2.5 Region of interest (ROI) definition

In the ROI analysis, the two sub‐conditions (one and three) of the action and ball conditions were considered separately; this analysis used a priori ROIs including the three levels of action observation network, as well as topographically maps of numerosity‐selective regions. The three occipito‐temporal regions of the Action observation network, the MT+, middle temporal gyrus (MTG), and occipito‐temporal sulcus (OTS) ROIs, were defined according to the studies of Ferri et al. (2015) and Orban et al. (2019). In parietal cortex, the phAIP, dorsal intraparietal sulcus medial/anterior (DIPSM/DIPSA) ROIs were defined according to Jastorff et al. (2010) and Georgieva, Peeters, Kolster, Todd, and Orban (2009). The region of the precentral sulcus in the frontal cortex was based on Jastorff et al. (2010). The different numerosity maps, NTO (“numerosity temporo‐occipital”), NPC1 (the first “numerosity postcentral”), NPC2 (the second “numerosity postcentral”), and NPC3 (the third “numerosity postcentral”), were defined according to Harvey and Dumoulin (2017) and Harvey et al. (2017). All maps were projected onto the flattened left and right hemispheres of the human PALS B12 atlas ([Van Essen, 2005] http://sumsdb.wustl.edu:8081/sums/directory.do?id=636032) using the Caret software package (Van Essen, Drury, Dickson, Harwell, and Anderson, 2001) [http://brainvis.wustl.edu/caret]). The number of voxels in the eight ROIs was as follows: left MT+ (*n* = 449), left MTG (*n* = 417), left OTS (*n* = 470), right MT+ (*n* = 306), left NPC1 (*n* = 152), left NPC2 (*n* = 86), left NPC3 (*n* = 203), and left phAIP (*n* = 419).

### 2.6 Univariate ROI analysis

To assess discrimination of the two numerosities, one and three, in the “Action” and “Ball” conditions, the percent MR signal changes relative to the active fixation were calculated for each voxel in the ROIs of each subject, for the four sub‐conditions, “1‐action,” “3‐actions,” “1‐ball,” and “3‐balls.” To calculate percent MR signal change, we applied to the nine runs a generalized linear model composed of 11 regressors: five for four experimental conditions (“1‐action,” “3‐actions,” “1‐ball,” and “3‐balls”) and the control condition (Fixation) and six for motion parameters. Individual voxels may exhibit preferences for either one or three OAs, which are averaged out by considering all voxels of a ROI together for the 3‐actions or 1‐action sub‐conditions. Hence, the first step to improve the sensitivity of the analysis consisted of recoding the action conditions into preferred and non‐preferred action numerosity. To avoid circularity, we introduced a cross‐validation procedure (Kriegeskorte, Simmons, Bellgowan, & Baker, 2009) using different runs to select the preferred action numerosity and to calculate the response to this condition. We split the nine runs into three runs for determining the preference of the voxel and the remaining six runs for evaluating responses of the voxel. As there are 84 ways to select three runs out of nine, we replicated the splitting procedure 84 times and averaged the results. In each replication, the action sub‐condition showing the largest (smallest) percent MR signal changes between the “1‐action” and “3‐actions” blocks in the three selection runs was considered the “preferred (non‐preferred) action” of the voxel and attributed the response calculated from the remaining six runs. The ball sub‐conditions were grouped into “preferred ball” and “non‐preferred ball” sets in a similar manner. The choice to use only a third of the runs for selection and the majority to evaluate the responses follows that of Serences, Saproo, Scolari, Ho, and Muftuler (2009), who used only one run out of four to select the preferred orientation of early visual cortex voxels (Serences et al., 2009). Control analyses showed that results were largely similar for the different ways of splitting the nine runs, ranging from a single run to eight runs for selection, and the remaining eight to a single run for calculation of the response. Once the responses of the voxels in the four recoded conditions were obtained, results were averaged across the voxels of each ROI. These average percent MR signal changes of the recoded conditions across subjects were analyzed using a two‐way repeated measure ANOVA for two factors: “action versus ball” and “preferred versus non‐preferred.” The threshold of statistical significance was controlled for multiple comparisons using Holm's method (Holm, 1979). For *n* distinct tests (in this case, *n* = 8, for eight different ROIs), the Holm method compares the *k*th smallest *p* value (for *k* = 1, 2, …, *n*) among the original *p* values with adjusted *p* value of .05/(*n*‐*k* + 1) until the *k*th smallest *p* value exceeds the adjusted *p* value. When the *k*th smallest *p* value is smaller than the adjusted *p* value, the *p* value is considered as statistically significant.

### 2.7 Selectivity of single voxels

To discriminate the numerosity of “action” or “ball,” the strength of the signal of each voxel of the ROI may have to contribute. Thus, in a second step to increase the analysis sensitivity, the single voxel contribution was assessed by comparing the differences between “preferred action” and “non‐preferred action” (labeled “dAction”) and between “preferred ball” and “non‐preferred ball” (labeled “dBall”) at single voxel level. For each voxel of a ROI, we calculated dAction and dBall in each subject using the values for the four recoded conditions obtained from cross‐validation. dAction and dBall can be considered a minimal measure of tuning for numerosity of actions and colored balls, respectively, emulating a study of orientation tuning of single voxels in early visual cortex (Serences et al., 2009). As we are searching for a selective tuning of single voxels for numerosity of actions and not colored balls, we required dAction to be larger than dBall, corresponding to an interaction between numerosity and attended feature at the single voxel level. Hence, we used the proportion of the voxels below the diagonal of the dAction‐dBall plane as metric of this interaction. Because cross‐validation approach involves splitting the data into two parts, it brings into play the reliability of the voxels, that is, the correlation of responses in two halves of the data. This reliability varies across brain region and across subjects (Tarhan & Konkle, 2020b) and can thus be a perturbing factor for small tuning effects of single voxels. To minimize the effect of unreliability, we averaged the percent MR signal change across subjects before comparing dAction and dBall, obtaining group‐averaged results, in following of Tarhan and Konkle (2020b). Indeed, comparing these two quantities for single subjects yielded much smaller effects. To reduce the effects of reliability in this latter group analysis, we tried to compare dAction and dBall in each replication of the cross‐validation, before averaging across replications, but this had only modest effects. The proportion of voxels below the diagonal was compared across ROIs using chi‐square tests when group‐averaging, or paired *t*‐tests when comparing across single subjects.

To assess the significance of the distribution of the voxels in the dAction‐dBall plane in the group‐averaged results, we performed a shuffling analysis, first at the individual subject level, and then the group level. The cross‐validated preferred and non‐preferred action, and preferred and non‐preferred ball activity for each voxel of a ROI, yielded two 2×N matrices for a ROI in each subject, where *N* is the number of voxels in the ROI. In each shuffle (*n* = 10,000), the links between the label and value of the two matrices were randomly reassigned, and the shuffled dAction‐dBall scatter diagram was calculated in each subject. Hence, we calculated for each shuffling a percentage of voxels below the diagonal, and the distribution of these percentages across the 10,000 replications for each ROI. These individual distributions followed a normal distribution, and they were averaged bin per bin (100 bins) across subjects, yielding the average shuffled distribution. The average shuffled distribution represents the null distribution to estimate significance of the actual proportion of voxels below the diagonal in each ROI, its *SD* being a measure of the distance of the actual proportion from chance.

### 2.8 Multivariate ROI analysis

In order to classify “1‐action” and “3‐actions” sub‐conditions, we employed multi‐voxel pattern analysis (MVPA) using percent MR signal change in eight ROIs as inputs to the pattern classifier (Norman, Polyn, Detre, & Haxby, 2006). The percent MR signal change relative to the active fixation for each voxel of the two sub‐conditions, “1‐action” and “3‐actions” (72 trials per each), were calculated in each subject in each ROI. Within those 72 trials, 64 trials from eight out of nine runs were used for training the classifier and the remaining eight trials from the left‐out run were used for testing. Using the two MR samples corresponding to the video presentation of each trial yielded a total of 128 training samples and 16 testing samples. Linear support vector machines (Cortes & Vapnik, 1995) and the LIBSVM software package (Chang & Lin, 2011) were used as the pattern classifier. Each of the nine runs was left out of the training set and used as test set, producing nine combinations. The training‐testing procedure was performed nine times in each subject and averaged. Finally, predicted accuracies were averaged across subjects in each ROI.

## 3 RESULTS

Accuracy, reaction time, and number of saccades for each of the four discrimination conditions are listed in Table 2. Accuracy averaged 97.1 ± 0.5% (mean ± *SE*) and did not differ across the four discrimination blocks (Table 2, repeated measure ANOVA, *F*_[3,60]_ = 0.658, *p* = .581). Reaction times also did not differ across the four discrimination blocks (Table 2, repeated measure ANOVA, *F*_[3, 60]_ = 1.237, *p* = .304). The number of saccadic eye movements averaged 4.3 ± 0.7 (mean ± *SE*) per minute during fixation trials. This number did not differ across the four different discrimination and fixation trials (Table 2, repeated measure ANOVA, *F*_[4, 76]_ = 1.246, *p* = .299).

**TABLE 2:** Accuracy, reaction time, and number of saccades (mean ± *SE*)

	Block			
	1‐action	3‐actions	1‐ball	3‐balls
Accuracy (%)	96.6 ± 0.9	97.3 ± 0.5	97.0 ± 0.6	97.6 ± 0.6
Reaction time (ms)	283.6 ± 62.8	313.6 ± 64.7	309.3 ± 63.9	299.1 ± 64.7
Number of saccades (per min)	4.7 ± 0.9	4.6 ± 0.8	4.8 ± 0.8	5.1 ± 0.9

### 3.1 Whole‐brain random‐effects group analysis

**FIGURE 2:**
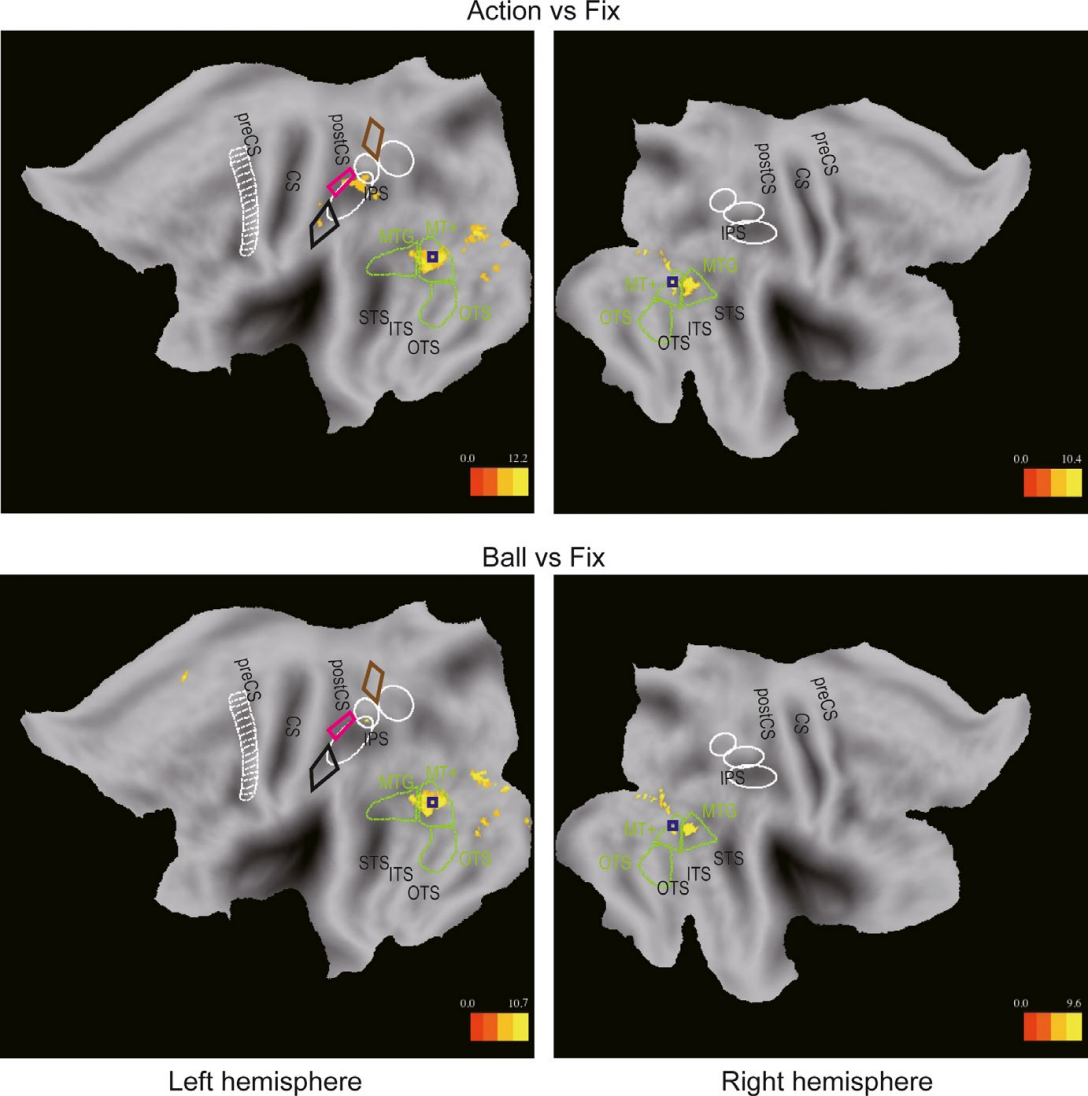
Statistical parametric maps showing significant voxels (*t* score color coded, see inset) for “Action” and “Ball” conditions compared to active fixation in the upper row and lower row, respectively. Left and right panels show left and right hemisphere flat maps, respectively (*p* < .05, FWE‐corrected). Blue squares indicate centers of bilateral NTO (Harvey & Dumoulin, 2017); white ellipses: confidence ellipses for the anterior intraparietal, dorsal intraparietal sulcus anterior, and dorsal intraparietal sulcus medial (from rostral to caudal); white ladder‐like outlines in the left hemisphere: premotor ROIs (Jastorff et al., 2010). Green outlines correspond to the MTG, MT+, and OTS ROIs (Orban et al., 2019), brown, magenta and black diamond outlines to NPC1, NPC2, and NPC3, respectively (Harvey et al., 2017). CS, central sulcus; IPS, intraparietal sulcus; ITS, inferior temporal sulcus; OTS, occipito‐temporal sulcus; post‐CS, postcentral sulcus; pre‐CS, precentral sulcus; STS, superior temporal sulcus

To evaluate which regions were activated in the “Action” condition (including “1‐action” and “3‐actions” blocks) and in the “Ball” condition (including “1‐ball” and “3‐balls” blocks), SPMs of the “Action” and “Ball” conditions, compared to active fixating baseline, were computed and are shown in Figure 2 at *p* < .05, FWE‐corrected level. In addition to the early visual cortex, the left MT+ region was strongly activated in both the “Action” and “Ball” conditions. Smaller activations of the right MT+ and MTG regions were also observed in both conditions. Bilaterally, the activation of the MT+ clearly overlapped with the center of the NTO (indicated by the blue rectangle in Figure 2), the topographic map of numerosity‐selective neurons located at the lateral temporal occipital junction (Harvey & Dumoulin, 2017). In the parietal regions, a remarkable difference between the “Action” and “Ball” conditions was observed in the caudal portion of the left phAIP region. The activation spread from the boundary between the caudal portion of the phAIP and ventral portion of the DIPSA to the middle of phAIP in the “Action” condition, but only included a small site on the boundary between the caudal phAIP and ventral DIPSA in the “Ball” condition. Thus, only this small “boundary” site was activated in both conditions. Moreover, there was a further difference in the regions dorsal and ventral to phAIP: NPC2 (magenta outlines in Figure 2) and NPC3 (black outlines in Figure 2), the second and third topographic maps of numerosity‐selective neurons located near the postcentral sulcus (Harvey & Dumoulin, 2017; Harvey et al., 2017), were activated in the “Action,” but not the “Ball” condition. SPMs did not show any significant site within the phAIP in the right hemisphere, despite the reversal of the actor's position in the visual field, which is the main source of parietal asymmetry in action observation (Ferri et al., 2015; Jastorff et al., 2010).

**FIGURE 3:**
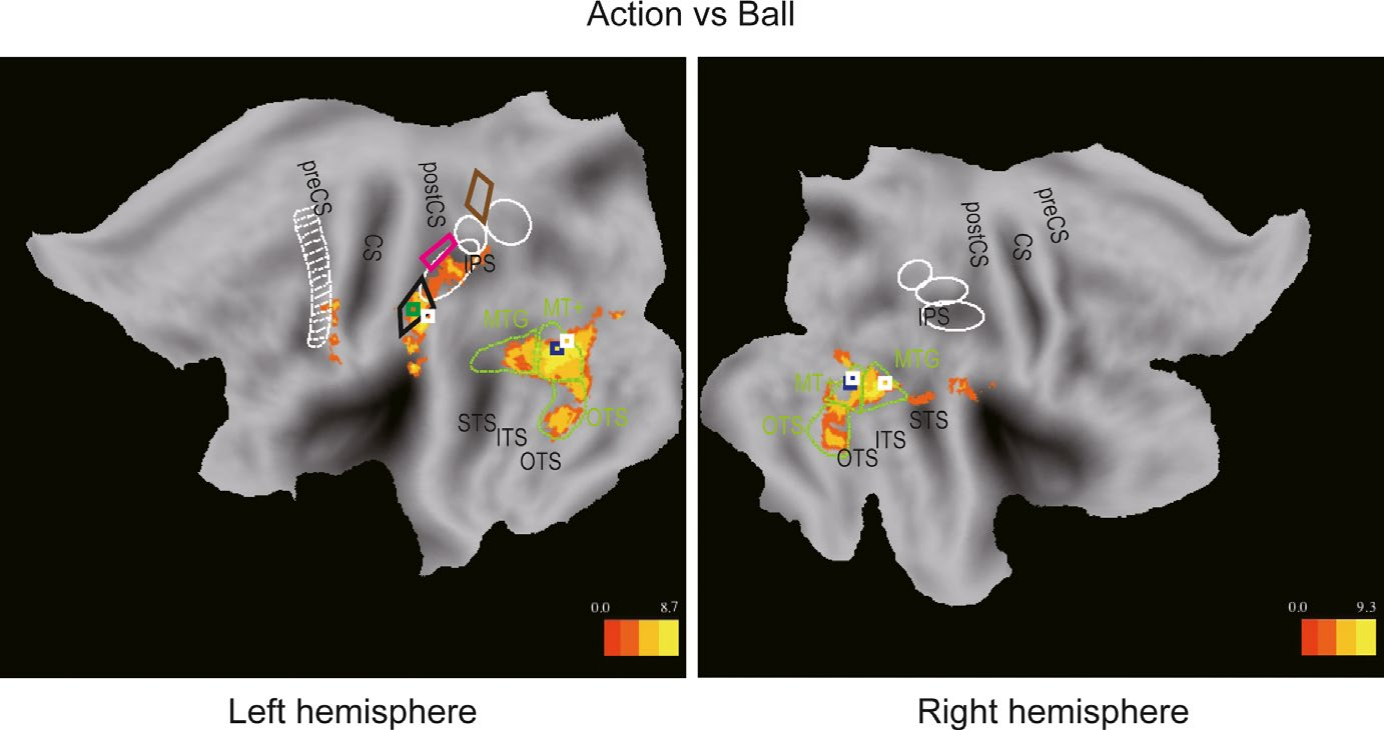
Statistical parametric maps showing significant sites for the “Action” condition compared to the “Ball” condition (*p* < .001, uncorrected; *p* < .05 at cluster level). Left and right panels show results from the left and right hemispheres, respectively. Green square indicates center of the left NPC3 (Harvey & Dumoulin, 2017). White squares indicate locations of local maxima that exceeded the threshold of FWE‐corrected *p* < .05 in the “Action” condition, compared to the “Ball” condition (Table 3). Same conventions as Figure 2

As a first step to identify regions in the parietal cortex that were selective for discriminating the number of performed actions, we identified the regions involved in processing the OAs by comparing the “Action” condition directly to the “Ball” condition. Four activation sites were identified by this main effect of attended feature at *p* < .05, FWE‐corrected level (listed in Table 3), and plotted at a threshold of *p* < .001, uncorrected, and cluster *p* < .05 FWE‐corrected level for illustrative purposes in Figure 3. Three of these four sites (Table 3) were located in occipito‐temporal cortex: two local maxima bilaterally inside the MT+ regions (indicated by a white rectangle in Figure 3), clearly overlapping the center of the NTO (indicated by a blue rectangle in Figure 3), and one local maximum in right MTG. The fourth local maximum (indicated by a white rectangle in Figure 3) was located on the border of left NPC3 (indicated by a black line in Figure 3), in fact not far from the center of NPC3 (Harvey & Dumoulin, 2017). No significant clusters (*p* < .05, FWE‐corrected level) were obtained in the reverse main effect of attended feature comparing the “Ball” condition to the “Action” condition, nor in the main effects of numerosity. Finally, the two interactions yielded no significant cluster at *p* < .05, FWE corrected, and only prefrontal clusters at a lower, descriptive level (*p* < .001 voxel level and *p* < .05 cluster level), one in each interaction.

**TABLE 3:** MNI coordinates (*x*, *y*, *z*), *Z* score of local maxima and cluster size (FWE *p* < .05 corrected) of main effect Actions vs Balls

(x, y, z)mm	Z score	Cluster size	Location
(46, −76, −4)	5.73	33	Right MT+
(48, −62, 6)	5.65	37	Right MTG
(−42, −82, −2)	5.63	136	Left MT+
(−60, −32, 40)	5.12	4	Left NPC3

Figure 3 shows that the action‐versus‐ball SPMs included at the descriptive level, bilateral occipito‐temporal regions, including the MT+, MTG, and OTS regions, as defined according to Ferri et al. (2015) and Orban et al. (2019). They further included an extension of the left NPC3 activation into phAIP caudally and supramarginal gyrus ventrally, and an elongated activation in the inferior portion of the left precentral sulcus region, partially overlapping with the premotor cortex ROI. No activations were observed in NPC1 (brown outlines in Figure 3) and NPC2 (magenta outlines in Figure 3). These results were generally consistent with those of a previous report (Orban et al., 2019), showing the recruitment of similar regions by a two‐alternative discrimination between OMAs. However, in the parietal cortex, activation was observed in the present study in both the left phAIP and neighboring NPC3 region, antero‐ventral to the phAIP. This latter region was not observed in the previous report by Orban et al. (2019), which compared discrimination of manipulative actions, to that of actors and target colors. The difference between the current experiment and that of Orban et al. (2019) was the need to process the numerosity of the OMAs and targets of the actions.

In conclusion, the random‐effects whole‐brain analysis revealed the regions involved in OMA processing (i.e. one main effect of attended feature), but failed to indicate regions where numerosity and attended feature interacted.

### 3.2 Univariate ROI analysis

**FIGURE 4:**
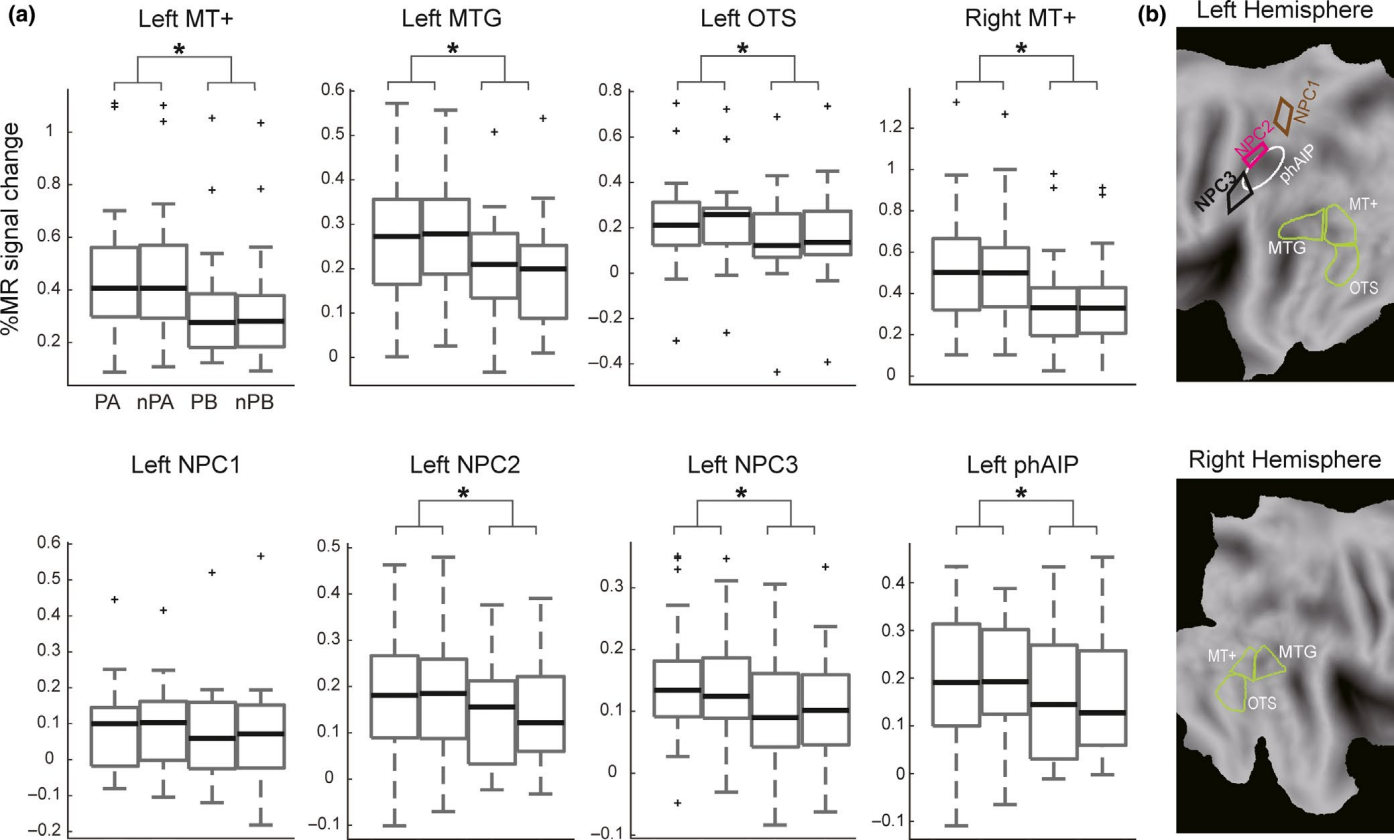
(a) Box plots of the percent MR signal changes (relative to fixation) in four reorganized sub‐conditions (preferred action, non‐preferred action, preferred ball, and non‐preferred ball) in the left MT+, left MTG, left OTS, right MT+, left NPC1, left NPC2, left NPC3, and left phAIP regions. The bold black lines mark indicates the median, and the top and bottom edges of the box represent the 75th and 25th percentiles. The "+" mark indicates outliers and whiskers extend to the most extreme data points without considering outliers. Asterisks indicate statistical significance corrected for multiple comparisons with Holm's method. PA, Preferred Action; nPA, non‐Preferred Action; PC, Preferred Ball; nPB, non‐Preferred Ball. (b) A flat map showing each ROI in the left hemisphere (upper panel) and in the right hemisphere (lower panel)

As NPC3, activated in the contrast action versus ball, is by definition a numerosity map (Harvey & Dumoulin, 2017), this region is a candidate for the processing of numerical information necessary to discriminate numerosity of manipulative actions. In addition to NPC3, NPC2 is another, be it less likely, candidate as this second postcentral numerosity map was activated in the “Action” condition but not the “Ball” condition compared to Fixation (Figure 2). For completeness, NPC1, in addition to NPC2 and NPC3, was also included as ROI to evaluate contribution of numerosity maps in parietal cortex. These three numerosity ROIs were compared to ROIs from the action observation network, active in the present study (Figure 3): left phAIP at parietal level, and left MTG, left OTS, and bilateral MT+ at the LOTC level. Individual voxels may exhibit preferences for either one or three OAs, which are averaged out by considering all voxels of a ROI together for the 3‐actions or 1‐action sub‐conditions. Indeed, SPMs revealed no significant activations at *p* < .05, FWE‐corrected level in the single effects “1‐action versus 3‐actions” and “1‐ball versus 3‐balls” in either direction. Hence, for each voxel the action sub‐condition showing the largest (smallest) value of the percent MR signal changes between the “1‐action” and “3‐actions” blocks was attributed to a “preferred (non‐preferred) action” group, using a cross‐validation procedure (see Materials and Methods). The ball sub‐conditions were grouped into “preferred ball” and “non‐preferred ball” sets in a similar manner. The average results for the recoded sub‐conditions are shown for the eight ROIs in Figure 4. The repeated‐measures ANOVA (with factors “action versus ball” and “preferred versus non‐preferred,” see Materials and Methods) confirmed that the percent MR signal change of the “Action” condition was significantly higher than that of the “Ball” condition in all ROIs, except NPC1. In contrast, none of the interactions between the two factors were significant after correction for eight comparisons, perhaps reflecting the weakness of the other main effect (Table 4). Thus, the recoding of the conditions was not enough to demonstrate a specific action numerosity effect, when averaging across voxels of ROIs. Hence, we turned to analysis of the individual voxels, rather than averaging over voxels of ROIs, following the work of Serences et al. (2009).

**TABLE 4:** Two‐way ANOVA of %MR signal change in univariate ROI analysis

Region	Main effect of Action vs Ball	Main effect of Preferred vs Non‐Preferred	Interaction
Left MT+	*F*_[1,20]_ = 50.958; *p* < .001*	*F*_[1,20]_ = 0.278; *p* = .604	*F*_[1,20]_ = 0.278;*p* = .604
Left MTG	*F*_[1,20]_ = 33.463; *p* < .001*	*F*_[1,20]_ = 0.006; *p* = .939	*F*_[1,20]_ = 1.614; *p* = .218
Left OTS	*F*_[1,20]_ = 18.865; *p* < .001*	*F*_[1,20]_ = 0.329; *p* = .573	*F*_[1,20]_ = 1.218; *p* = .283
Right MT+	*F*_[1,20]_ = 43.692; *p* < .001*	*F*_[1,20]_ = 2.22; *p* = .152	*F*_[1,20]_ = 0.240; *p* = .630
Left NPC1	*F*_[1,20]_ = 3.703; *p* = .069	*F*_[1,20]_ = 0.085; *p* = .773	*F*_[1,20]_ = 0.417; *p* = .526
Left NPC2	*F*_[1,20]_ = 7.165; *p* = .014*	*F*_[1,20]_ = 0.239; *p* = .630	*F*_[1,20]_ = 0.536; *p* = .473
Left NPC3	*F*_[1,20]_ = 23.475; *p* < .001*	*F*_[1,20]_ = 0.024; *p* = .879	*F*_[1,20]_ = 3.115; *p* = .093
Left phAIP	*F*_[1,20]_ = 8.632; *p* = .008*	*F*_[1,20]_ = 1.029; *p* = .322	*F*_[1,20]_ = 0.145; *p* = .708

### 3.3 ROI analysis: selectivity of single voxels

**FIGURE 5:**
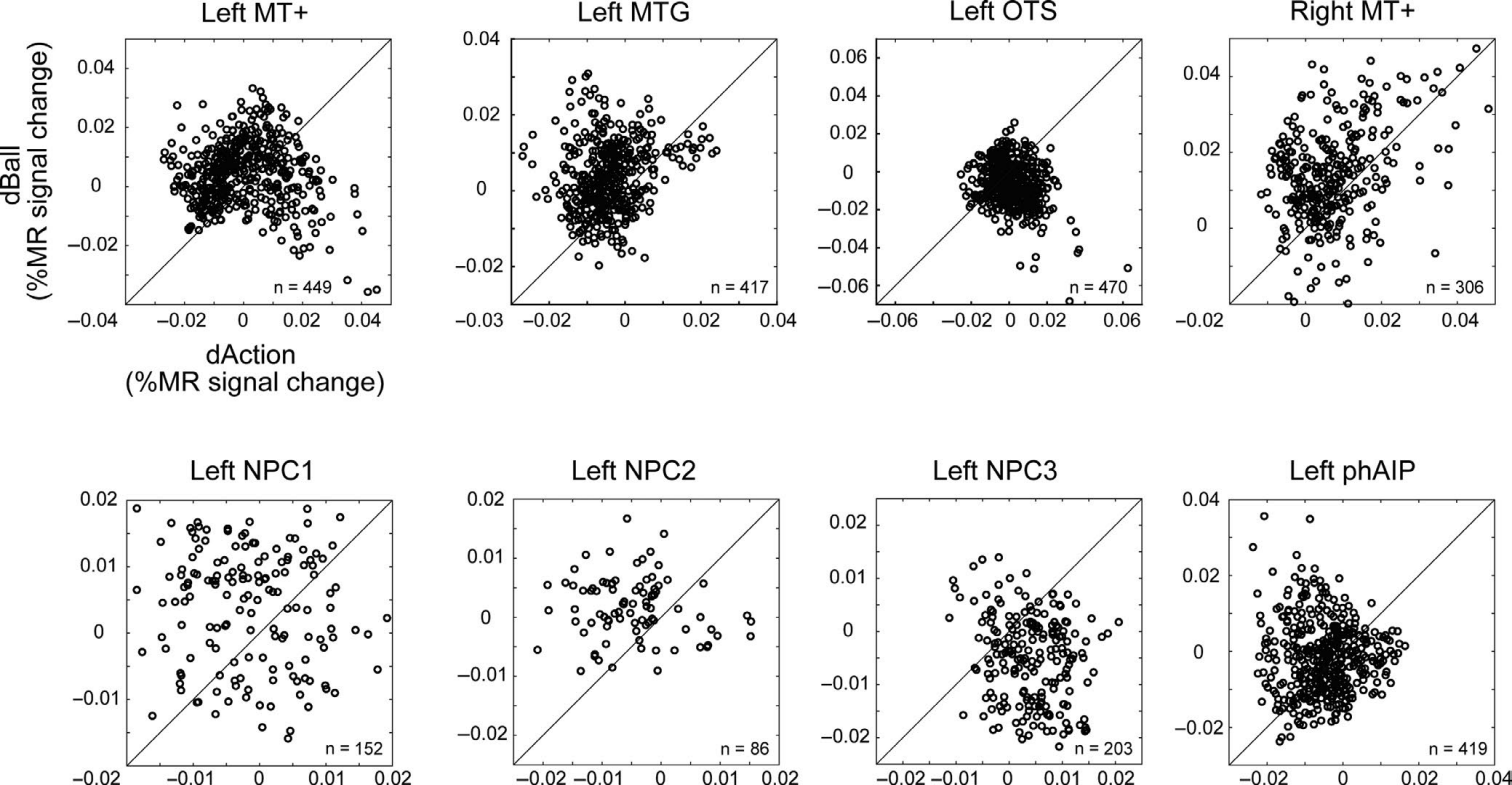
The scatter diagram of the percent MR signal change of “dAction” (abscissa) and “dBall” (ordinate) in the eight regions. Each point corresponds to a voxel. The diagonal line indicates the points where dAction is equal to dBall. The proportion of voxels below the diagonal was significantly larger in NPC3 than in each of the seven other ROIs (Table 5)

To assess the contribution of individual voxels to the discrimination of action numerosity, the differences between “preferred action” and “non‐preferred action” (labeled “dAction”) and between “preferred ball” and “non‐preferred ball” (labeled “dBall”) were compared at single voxel level. A systematic difference between “dAction” and “dBall” in favor of “dAction” should result in more than half the voxels being located below the diagonal of the dBall–dAction. This would indicate that the region specifically contributes to discriminating the numerosity of actions. To counteract the effects less reliable voxels, we started with the group average analysis, of which the scatter diagrams of “dAction” and “dBall” for the eight ROIs are shown in Figure 5. In these plots, each dot corresponds to a voxel and the metric for selectivity for action numerosity is the number of voxels below the diagonal (see Materials and Methods). In fact, the scatter diagrams visualize the interaction between numerosity and attended feature at the single voxel level: Concentration of the voxels near the diagonal line indicates an absence of interaction, as mentioned before, aggregation of the voxels below the diagonal indicate selectivity of numerosity processing for OMAs, while aggregation of voxels above the diagonal indicate selectivity of numerosity for the colored ball numerosity. Relatively, few voxels were located below the diagonal line of the scatter diagrams in NPC1 (34%) and NPC2 (21%). In bilateral MT+, left MTG, and left phAIP, again less than half the voxels were located below the diagonal (Table 5). On the contrary, as expected, many more voxels were distributed below the diagonal line (169 out of 203 voxels, 83%) than above in NPC3. Also left OTS showed a larger number of voxels below the diagonal, although clearly less than NPC3 (308 out of 470, 66%). Paired *t*‐tests confirmed that in all ROIs, the differences between dAction and dBall were significant (Table 5), but only in NPC3 and OTS were the differences in favor of dAction. Importantly, the number of voxels below the diagonal was significantly larger in NPC3 compared (in pairwise χ^2^ tests) to all other seven ROIs, including OTS (Table 5).

**TABLE 5:** Group average analysis: Percent voxels below the diagonal

Region	Total number of voxels	% below Diagonal	Paired *t* test (dAct–dBall) [*t*‐value, *df*,*p* value]	χ2 test (NPC3 vs other ROIs) [χ2 (*df* = 1), *p* value]
Left MT+	449	30.06	[−5.4134, 448, <.000*]	[158.89,<.0001*]
Left MTG	417	23.74	[−14.475, 416, <.000*]	[197.03, <.0001*]
Left OTS	470	65.53	[9.904, 469, <.000*]	[21.56, <.0001*]
Right MT+	306	28.10	[−9.017, 305, <.000*]	[148.46, <.0001*]
Left NPC1	152	34.21	[−5.050, 151, <.000*]	[88.96, <.0001*]
Left NPC2	86	20.93	[−6.053, 85, <.000*]	[102.74, <.0001*]
Left NPC3	203	83.25	[12.134, 202, <.000*]	N/A
Left phAIP	419	45.34	[−5.039, 418, <.000*]	[80.51, <.0001*]

* Statistical significance, after correction with Holm's method.

**FIGURE 6:**
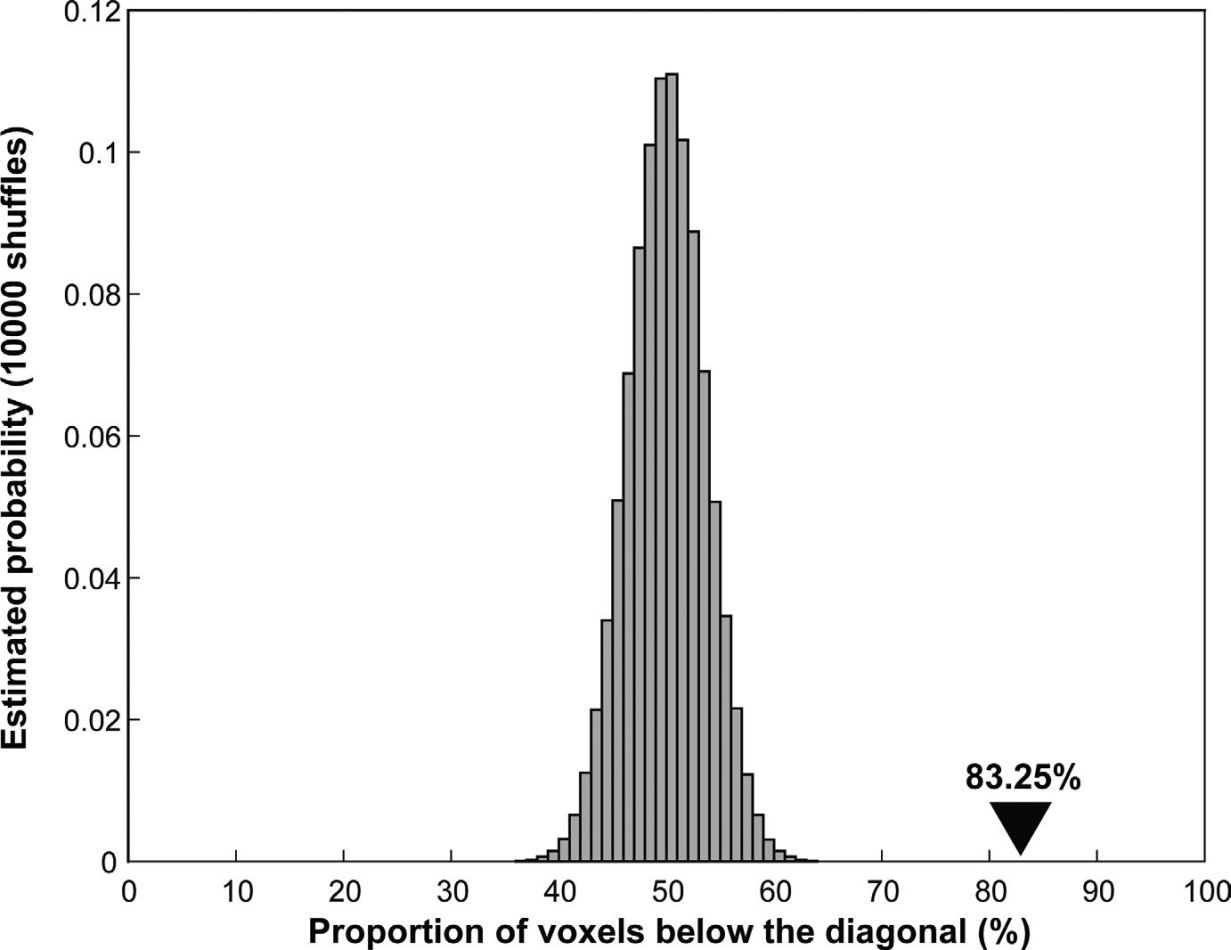
The frequency histogram of the proportion of voxels below the diagonal (abscissa) in NPC3 obtained from 10,000 shuffles displayed as estimated probability (ordinate). The proportion of voxels below the diagonal was calculated for each of 10,000 shuffles in each subject and averaged across subjects. The distribution follows normal distribution (mean and *SD* see Table 6). The black arrow head indicates the actual value from the 203 single voxels in NPC3 (Figure 5) that deviated significantly (*p* < .001) from the shuffled distribution

Shuffling analysis, breaking the link between labels and values of the voxel‐condition matrices (see Materials and Methods) repeated 10,000 times, produced shuffled distributions which followed a normal distribution. The average shuffled distribution in NPC3 is shown in Figure 6, while the mean and *SD* of the average shuffled distributions in the eight ROIs are listed in Table 6. The actual percentage of voxels below the diagonal in NPC3 was more than 4 *SD* removed from the mean of the shuffled distribution (Figure 6), indicating that it differed significantly (*p* < .001) from chance. In most other ROIs, except phAIP, the deviation of the actual % from the mean of the shuffled distribution exceeded 3SD indicating again a significant (*p* < .0026) difference from chance. In all these ROIs, except OTS, the actual value deviated to the left of the chance distribution, indicating significantly more voxels above the diagonal.

**TABLE 6:** The mean (%) and *SD* (%) of shuffled distributions

Region	Mean (%)	*SD* (%)	Distance between group average results and shuffled mean (in *SD*)
Left MT+	50.012	2.414	−8.265
Left MTG	49.989	2.452	−10.705
Left OTS	50.017	2.311	6.713
Right MT+	50.026	2.801	−7.828
Left NPC1	50.046	3.980	−3.979
Left NPC2	50.047	5.348	−5.444
Left NPC3	50.009	3.575	9.298
Left phAIP	50.010	2.454	−1.903

Thus, at the group‐average level NPC3 stood out as hosting a majority of voxels that showed a larger differential activity for the preferred numerosity of OAs than color of balls. To evaluate how well this analysis applied to the group of subjects, the proportion of voxels below the diagonal was calculated in each ROI and each subject, and results averaged across subjects. These group results (Table 7) emulated the group‐average results (Table 6). Indeed, NPC3 (56%) and, to a lesser degree, OTS (53%) were the only ROIs whose average ratio exceeded 50% (Table 7), with the remaining 6 ROIs showing average proportions between 45% and 47%. One‐tailed *t*‐tests showed that only NPC3 showed a trend toward significance (Table 7). The differences between ROIs were smaller at the group than the group‐average level, and only the difference between NPC3 and left MTG, left MT+, and NPC2 showed statistical significance at uncorrected level (Table 7). In an effort to reduce the effects of less reliable voxels, we calculated the % below the diagonal for each replication of the cross‐validation, and then average the numbers across replications to obtain the number of voxels below the diagonal in each subject. This refined analysis returned similar effects to the standard group analysis, but, as intended, the variability across subjects was reduced. Hence, the one‐tailed *t*‐test in NPC3 reached significance at uncorrected level (*t* = 2.0907, *df* = 20, *p* = .0248), and the paired *t*‐test between NPC3 and MTG reaches significance at corrected level (*t* = 3.643, *df* = 20, *p* = .002).

**TABLE 7:** Group analysis: the proportion of voxels below diagonal

Region	Mean (%)	*SD* (%)	One‐tailed *t* test compared to 50% [*t*‐value (*df* = 20), *p* value]	Paired *t* test (NPC3 vs other ROIs) [*t*‐value (*df* = 20), *p* value]
Left MT+	45.70	13.97	*t* = −1.411 *p* = .087	*t* = 2.234 *p* = .037
Left MTG	46.68	14.64	*t* = −1.038 *p* = .156	*t* = 2.362 *p* = .028
Left OTS	53.26	15.08	*t* = 0.991 *p* = .167	*t* = 0.699 *p* = .493
Right MT+	45.21	23.70	*t* = −0.927 *p* = .183	*t* = 1.624, *p* = .120
Left NPC1	47.18	22.32	*t* = −0.579 *p* = .285	*t* = 1.546 *p* = .138
Left NPC2	44.96	22.72	*t* = −1.016 *p* = .161	*t* = 2.114 *p* = .047
Left NPC3	55.95	16.21	*t* = 1.6812 *p* = .054	N/A
Left phAIP	47.04	21.54	*t* = −0.630 *p* = .268	*t* = 1.649 *p* = .115

### 3.4 MVPA ROI analysis

A MVPA using percent MR signal change in NPC3 to discriminate “1‐action” and “3‐actions” was also conducted. The accuracy (48.0% across the 21 subjects) did not exceed chance level of 50%. Similar results were obtained in the other 7 ROIs (Table 8). Thus, the distribution of the 1‐action and 3‐action activations could not reliably be segregated into two clusters, in the voxel hyperspace, even in NPC3. It is important to note that MVPA decodes, at the group level, action numerosity from the pattern of activation in a set of voxels, while the single voxel analysis compared dAction and dBall activation, only counting selective voxels, and obtaining significant results at the group‐average level, with only a trend at the group level.

**TABLE 8:** Results of a multi‐voxel pattern analysis

Region	Mean accuracy (%)	*SD* (%)	Chance level (%)
Left MT+	49.2	2.8	50
Left MTG	48.8	3.6	50
Left OTS	48.3	2.9	50
Right MT+	49.0	3.6	50
Left NPC1	47.8	2.8	50
Left NPC2	49.3	3.7	50
Left NPC3	48.0	2.6	50
Left phAIP	49.8	3.4	50

## 4 DISCUSSION

Our results bore out the predictions: One of the candidate parietal regions, NPC3, was found to be involved in the processing of OMA numerosity. However, this role of NPC3 was not revealed by the SPM of the interaction between numerosity and attended feature, but by a novel group‐averaged analysis of the differential activation in single voxels of selected ROIs. Indeed, NPC3 was significantly more active in action than in ball discrimination (Figures 3 and 4), but more importantly, the difference in activation of individual NPC3 voxels between the better of the two numerosities and the other was significantly larger for action than for ball discrimination (Figure 5). This contrasted with the neighboring phAIP and NPC2 regions which shared the first, but not the second, more revealing characteristic of NPC3. However, one of the LOTC regions, left OTS, showed similarities with the NPC3, although the selectivity for action numerosity over colored ball numerosity was significantly lower in OTS than in NPC3. Furthermore, the contrast between action and ball discrimination revealed the activation of several LOTC regions, including two more numerosity maps: bilaterally NTO. These two maps, as the other LOTC region, left MTG, showed the opposite selectivity compared to NPC3, being more selective for discriminating colored ball than action numerosity.

### 4.1 Effects of attentional modulation

Behavioral performance (e.g., accuracies, reaction times, and numbers of saccades) did not differ across the four different discrimination blocks (Table 2). Moreover, identical visual stimuli were presented in the four discrimination conditions. Hence, it is unlikely that the differences between the activation maps obtained for “Action” and “Ball” conditions, compared to active fixation, can be attributed to behavioral performances or low‐level visual responses. In contrast, they likely reflect attention to different features: identity of the OA (push or flick) or color of the target (white or orange). Although the tasks were easy, they still required the subject to process completely different aspects of the stimuli. This interpretation is in agreement with earlier reports using a similar paradigm (i.e., manipulating featural attention within a constant stimulus), thus revealing attentional modulation of occipito‐temporal cortex (Cant & Goodale, 2007), parietal cortex (Orban et al., 2019), and face‐selective cortical regions (Chiu et al., 2011). These findings are further supported by the observation that featural attention affects selective hemodynamic activity in fMRI (Cukur, Nishimoto, Huth, & Gallant, 2013; Stoppel et al., 2011), as well as by many neurophysiological experiments in non‐human primates (Bisley & Goldberg, 2010; Freedman & Ibos, 2018; Treue & Martinez Trujillo, 1999). Indeed, these latter studies have indicated that featural attention modulates the gain of tuning functions, and recent single cell studies in AIP and its human counterpart have demonstrated neuronal tuning for OMAs (Aflalo et al., 2020; Lanzilotto et al., 2019). It is theoretically true that once action/ball is repeated more than once in the video, participants have sufficient information to solve the task. However, we asked the participant to direct careful attention to the number of actions/colored balls in the videos, and at any rate subjects had to discriminate action numerosity, either one and three, or at least, one and more than one.

### 4.2 Recruitment of a parietal component of the action observation network: phAIP

The phAIP region was activated in the contrast *action* versus *ball* discrimination, at a descriptive level in the whole‐brain analysis, and at corrected level in the ROI analysis. The present finding is in close agreement with our earlier study (Orban et al., 2019) in which phAIP was activated bilaterally when subjects attended to OMAs, rather than to the gender of the actors or color of the targets. These findings, together with the single cell studies mentioned above (Aflalo et al., 2020; Lanzilotto et al., 2019), and a recent stereo‐EEG study (Platonov et al., 2020) support the view that the identity of the OMAs is processed in this region. One difference between the two fMRI studies is that activation in phAIP was observed only in the left hemisphere in the current study. This difference is likely to originate from the differences in cognitive operations required by the tasks used in the two experiments. In the previous study, the action, color of the target, and gender of the actor were assessed for single items, while the current task required first to ascertain the OA identity and then evaluate the numerosity of the identified actions. This additional step may have forced the action information to be channeled into the NPC3 region, rather than being transmitted directly to decision processing regions. As NPC3 operates with a hemispheric bias to the left, for reasons given below, this may also have biased the afferent phAIP activation.

Within phAIP, the activation, although slightly different when the “Action” condition was compared to active fixation or to the “Ball” condition, occupied mainly the caudal half of the ROI. This was also true in Experiment 2, but not Experiment 1 of the Orban et al. (2019) study. These subtle displacements of the activation sites within phAIP may reflect the observed exemplars used as the discriminanda in the different experiments. Indeed, it has been reported that the rostral and caudal portions of the phAIP distinguish OAs that moved objects toward or away from the subject, thereby categorizing actions as positive or negative (Jastorff et al., 2010). Both actions used in the present study, moved the balls away from the actor, as was the case for one of the actions used in experiment 2 of Orban et al. (2019), while in Experiment 1 of that study, both actions moved the objects toward the actor. Thus, the exemplars used in action discrimination may account for the small differences in activation location within phAIP. Several studies have also provided clear indications that while OA identity was processed in PPC (Jastorff et al., 2010), the effectors used in the OA were processed in the premotor cortex (Fabbri, Stubbs, Cusack, & Culham, 2016; Fujii, Hihara, & Iriki, 2008; Jastorff et al., 2010). The difference in effectors between push and flick (i.e., the hand and index finger) may thus account for the left precentral activation in the *action* versus *ball* discrimination contrast of the present study, as it did in experiment 2 of Orban et al. (2019).

### 4.3 Discriminating numerosity of observed actions

Third numerosity postcentral sulcus map was recruited when the “Action” condition was compared to the “Ball” condition (Table 3). This indicates that this numerosity map processes OAs, and thus might integrate numerosity and OMAs. This failed to be supported in the whole‐brain or the univariate ROI analysis by an interaction between numerosity and attended feature, as originally planned. However, further analyses involving recoding the action conditions and assessing the selectivity of individual voxels suggested that NPC3 processes the numerosity of OAs rather than that of the targets. This interpretation is in accordance by the large number of voxels for which “dAction” exceeded “dBall” in the NPC3 scatter plot (Figure 5). This number was significantly larger in NPC3 than in the other seven ROIs. While NPC1 showed no significant difference between “Action” and “Ball” condition in the percent MR signal change (Figure 4), NPC2 did so, though SPM only revealed the activation in NPC2 when “Action” was compared to “Fixation” (Figure 2), not to “Ball” condition (Figure 3). However, the scatter diagram (Figure 5) indicates that NPC2 does contribute little or nothing to the processing of OMA numerosity. Thus, NPC3 is the only parietal numerosity map for which our results suggest a role in the processing of action numerosity. It is important to note that these results were obtained at the group‐average level, and only partially generalized to the group analysis of single subject data (Table 7). One likely source of the difference in statistical power of these two analyses is the lack of reliability of a number of voxels, called into play by the data splitting required by the cross‐validation procedure. Reliability of voxels, which has only recently been recognized (Tarhan & Konkle, 2020a), may have been weakened by the duration of single trials (8.1 s), reducing the number of trials collected per condition in each subject. As reliability varies widely across brain regions and subjects (Tarhan & Konkle, 2020b), the most straight forward way to reduce its effects is to perform a group average as we did, in following of Tarhan and Konkle (2020b). Thus, while our results suggest that NPC3 is the only of the three parietal numerosity maps involved in processing numerosity of OMAs, further work is needed to demonstrate that this holds in group analyses, which will require testing explicitly the reliability of voxels (by scanning many conditions) and scanning many subjects to retain enough subjects with substantial fractions of reliable voxels in the relevant ROIs.

Third numerosity postcentral sulcus map, however, was not the only ROI in which the number of voxels below the diagonal exceeded 50%: Left OTS, an LOTC region, also displayed this pattern, although more weakly than NPC3. OTS is likely the homologue of the rostral part of the lower bank of monkey STS (Jastorff, Popivanov, Vogels, Vanduffel, & Orban, 2012), and neurons in this region are sensitive to the relationship between the movements of an object, target of the action, and those of the hand, performing the action [so‐called agent‐object interaction neurons, (Perrett et al., 1989)]. These relationships differ between the two OMAs (flick and push), presented in different numbers in the two action conditions. This small stimulus difference may receive more attention during action processing than ball processing and hence cause slightly larger differences in activation between the two action conditions than the two ball conditions. These signals related to hand–target relationships may underlie the transitivity category effects described in LOTC by Wurm et al. (2017). In the monkey, this rostral part of the lower bank of STS region projects to AIP (Lanzilotto et al., 2019), and the visual signals related to hand–target relationships probably enter into the definition of the visual identity of OMAs (combination of the observed goal of the action and perceived body movements bringing about this result) at that level (Lanzilotto et al., 2020). AIP neurons are tuned to OMAs (Lanzilotto et al., 2019), and if those tuned to flick and push are equal in number, little difference between the two action conditions is expected. The present results (Tables 5 and 7) suggest that the human homologue, phAIP (Orban, 2016), operates as hypothesized for monkey AIP. They further suggest that the visual OMA identity signals travel from phAIP to neighboring NPC3 for the processing of numerosity of OMAs. This fits with our finding that NPC3 has significantly more voxels below the diagonal than OTS.

That processing of OMA numerosity recruits NPC3 in the left but not the other hemisphere is in accordance with an earlier report indicating that lower numerosity, approximately less than three, was represented in the left hemisphere, while larger numerosities were represented in the right hemisphere (Harvey & Dumoulin, 2017). The local maximum in the left parietal cortex obtained in the current experiment was slightly displaced from the NPC3 center in the study by Harvey and Dumoulin (2017). This may be due to stimulus differences: small shapes presented simultaneously versus actions displayed sequentially. It is noteworthy that NPC3 occupies the caudal part of cyto‐architectonic region PFt (Caspers et al., 2006), the rostral part of which overlaps with anterior supramarginal gyrus, the region involved in tool action observation (Peeters, Rizzolatti, & Orban, 2013; Peeters et al., 2009). Thus, it is plausible that NPC3 processes the numerosity of OMAs performed with either natural effectors or artificial implements.

The activation of NPC3 in a task requiring the processing of numerosity is in agreement with a large body of imaging studies involving human parietal cortex in comparisons of numerals and non‐symbolic numerosity (Eger et al., 2009; Gobel, Johansen‐Berg, Behrens, & Rushworth, 2004; Piazza, Izard, Pinel, Le Bihan, & Dehaene, 2004; Piazza, Pinel, Le Bihan, & Dehaene, 2007; Pinel, Piazza, Le Bihan, & Dehaene, 2004). But, our results seem to contradict a recent report stating that the parietal cortex does not contribute to the estimation of sequential numerosity (Cavdaroglu & Knops, 1991). However, the tasks used in the two experiments differed in several ways. First, instructions were given prior to the trial in our study; second, subjects actively discriminated the numerosity of OAs presented sequentially rather than of simple dots presented simultaneously; and third, numbers were very small (1–3) in the present study, but exceeded four in the earlier study. Several single cell studies have shown that numerosity of sequential items is represented in monkey parietal cortex: The numerosity of motor acts executed by the monkey (Sawamura, Shima, & Tanji, 2002, 2010) as well as the numerosity of small shapes (Nieder, Diester, & Tudusciuc, 2006). In both cases, the numerosities tested were small, suggesting the numerosity range might be the critical factor to obtain parietal activation. Furthermore, several behavioral studies (Anobile, Turi, Cicchini, & Burr, 2012; Piazza, Fumarola, Chinello, & Melcher, 2011; Revkin, Piazza, Izard, Cohen, & Dehaene, 2008) have suggested that subitizing and enumeration of larger numbers use different mechanisms, although neuronal differences are not yet clear (Cai et al., 2018; Piazza, Mechelli, Butterworth, & Price, 2002). Also, it has been argued that numerosity maps revealed by Harvey and colleagues do not reflect the processing of numerosity per se but the processing of a conjunction of sensory cues that correlate with numerosity (Gebuis, Gevers, & Cohen Kadosh, 2014). Hence, we cannot exclude that several of the factors characterizing numerosity stimuli interact in PPC. Numerosity can be presented through various sensory channels (visual, auditory, tactile) or even through motor acts, items may be presented simultaneously or sequentially, differ in nature (dots, beeps, OAs), and span different ranges of numerosity. Only part of the numerosity space has been explored so far, most studies using visual dots presented simultaneously. Furthermore, some parts of this space may be only poorly covered: For example, observed or executed actions are typically sequential as an actor usually performs only one action at a time (except, e.g., juggling). Also, actors repeating identical actions are increasingly unlikely as the number of repetitions increases. Beyond vision, the auditory channel is suitable for communicative actions, yet provides little information about actions of others (stepping on a hard surface, or crumbling a leaf being some of the exceptions). Similarly, tactile information about others' actions is limited to interactions between subjects. Hence, visual observation of a few sequential actions is the prototypical case for numerosity of others' actions, and the present study used such stimuli.

### 4.4 Recruitment of bilateral NTO maps in action and ball discrimination

While in the previous study (Orban et al., 2019) only a single PPC region was revealed by comparing OA to color and gender discrimination, the MT+ regions, as well as other LOTC regions, appeared bilaterally in the contrast *action* versus *ball* discrimination in the present study (Figure 3). One reason may be the different cognitive requirements in the two studies, as numerosity of the attended feature had to be assessed in the present study but not the earlier one. This fits with the perfect overlap of the MT+ activation with the NTO maps. The NTO map has been suggested as a region processing the numerosity of motions and objects (Harvey & Dumoulin, 2017), and MT+ has also been activated by sequentially presented numerosity in other studies (Cavdaroglu, Katz, & Knops, 2015; Cavdaroglu & Knops, 1991). A second reason may be the differences in video clip duration. The current experiment used a long video of 5.4 s during which motion information was continuously present as balls continued to roll on the table after the action, whereas the prior experiment used short 1.5‐s videos, centered on the action itself. Thus, the current experiment involved more computation of visual motion. As these motion computations were more relevant for action than color (of ball) discrimination, the stimulus effect may have been amplified in action relative to ball discrimination. The difference in video duration may also account for the additional activation in the action versus ball contrast of two left LOTC regions.

Although the MT+ regions were more activated by the action than the ball conditions, at the single voxel level most voxels were located above the diagonal of the dAction‐dBall plane, indicating that dBall was significantly larger than dAction. These differences in relative activity within the ball and action conditions may simply indicate that at the level of the NTO numerosity maps, only visual signals related to motion events, and not more complex signals related to action identity, are available. A similar explanation may apply to left MTG, which has no known numerosity map, but may further process signals received from MT+. The MT+ results in fact fit with our view that visual identity of OAs becomes available only at the parietal level (Orban, 2018). The single voxel results in NPC1 and NPC2 (Figure 5) can then be understood as indicating that visual identity of action signals become available only at more rostral levels in PPC, as we have proposed above. Further work is needed to understand the exact role of NTO and the other numerosity maps in covering the numerously space, discussed above.

## 5 CONCLUSION

The current study suggests that one of the numerosity maps, NPC3 in the parietal cortex, is devoted to the processing the numerosity of OMAs, possibly using input regarding OMA identity, provided by neighboring phAIP.

## CONFLICT OF INTEREST

The authors declare no conflict of interests.

### Peer review

The peer review history for this article is available at https://publons.com/publon/10.1111/ejn.14930

## AUTHOR CONTRIBUTIONS

HS and GAO designed the study. HS and DC performed the experiments and obtained the data. HS and BAU analyzed the data. HS, BAU, and GAO made the figures and wrote the manuscript. All authors read and approved the final version of the manuscript.

## Data Availability

All raw data are available from the authors upon request.
